# Transformation of primary care during the COVID-19 pandemic: experiences of healthcare professionals in eight European countries

**DOI:** 10.3399/BJGP.2020.1112

**Published:** 2021-07-07

**Authors:** Marta Wanat, Melanie Hoste, Nina Gobat, Marilena Anastasaki, Femke Böhmer, Slawomir Chlabicz, Annelies Colliers, Karen Farrell, Maria-Nefeli Karkana, John Kinsman, Christos Lionis, Ludmila Marcinowicz, Katrin Reinhardt, Ingmarie Skoglund, Pär-Daniel Sundvall, Akke Vellinga, Theo JM Verheij, Herman Goossens, Christopher C Butler, Alike van der Velden, Sibyl Anthierens, Sarah Tonkin-Crine

**Affiliations:** Nuffield Department of Primary Care Health Sciences, University of Oxford, Oxford, UK.; Department of Family Medicine and Population Health, University of Antwerp, Antwerp, Belgium.; Nuffield Department of Primary Care Health Sciences, University of Oxford, Oxford, UK.; Clinic of Social and Family Medicine, Faculty of Medicine, University of Crete, Crete, Greece.; Rostock University Medical Centre, Rostock, Germany.; Department of Family Medicine, Medical University of Bialystok, Bialystok, Poland.; Department of Family Medicine and Population Health, University of Antwerp, Antwerp, Belgium.; School of Medicine, National University of Ireland, Galway, Ireland.; Clinic of Social and Family Medicine, Faculty of Medicine, University of Crete, Crete, Greece.; Expert social and behaviour change, European Centre for Disease Prevention and Control (ECDC), Solna, Sweden.; Clinic of Social and Family Medicine, Faculty of Medicine, University of Crete, Crete, Greece.; Department of Obstetrics, Gynaecology and Maternity Care, Medical University of Bialystok, Bialystok, Poland.; Institute of General Practice, Rostock University Medical Centre, Rostock, Germany.; General Practice/Family Medicine, School of Public Health and Community Medicine, Institute of Medicine, Sahlgrenska Academy, University of Gothenburg, Sweden.; General Practice/Family Medicine, School of Public Health and Community Medicine, Institute of Medicine, Sahlgrenska Academy, University of Gothenburg, Sweden.; School of Medicine, National University of Ireland, Galway, Ireland.; Julius Center for Health Sciences and Primary Care, University Medical Center Utrecht, Utrecht, the Netherlands.; Laboratory of Medical Microbiology, Vaccine & Infectious Disease Institute, University of Antwerp, Antwerp, Belgium.; Nuffield Department of Primary Care Health Sciences, University of Oxford, Oxford, UK.; Julius Center for Health Sciences and Primary Care, University Medical Center Utrecht, Utrecht, the Netherlands.; Department of Family Medicine and Population Health, University of Antwerp, Antwerp, Belgium.; Nuffield Department of Primary Care Health Sciences, University of Oxford, Oxford, UK.

**Keywords:** primary health care, semi-structured interviews, qualitative research, COVID-19

## Abstract

**Background:**

Primary care has a crucial role in responding to the COVID-19 pandemic as the first point of patient care and gatekeeper to secondary care. Qualitative studies exploring the experiences of healthcare professionals during the COVID-19 pandemic have mainly focused on secondary care.

**Aim:**

To gain an understanding of the experiences of European primary care professionals (PCPs) working during the first peak of the COVID-19 pandemic.

**Design and setting:**

An exploratory qualitative study, using semi-structured interviews in primary care in England, Belgium, the Netherlands, Ireland, Germany, Poland, Greece, and Sweden, between April and July 2020.

**Method:**

Interviews were audiorecorded, transcribed, and analysed using a combination of inductive and deductive thematic analysis techniques.

**Results:**

Eighty interviews were conducted with PCPs. PCPs had to make their own decisions on how to rapidly transform services in relation to COVID-19 and non-COVID-19 care. Despite being overwhelmed with guidance, they often lacked access to practical training. Consequently, PCPs turned to their colleagues for moral support and information to try to quickly adjust to new ways of working, including remote care, and to deal with uncertainty.

**Conclusion:**

PCPs rapidly transformed primary care delivery despite a number of challenges. Representation of primary care at policy level and engagement with local primary care champions are needed to facilitate easy and coordinated access to practical information on how to adapt services, ongoing training, and access to appropriate mental health support services for PCPs. Preservation of autonomy and responsiveness of primary care are critical to preserve the ability for rapid transformation in any future crisis of care delivery.

## INTRODUCTION

Since December 2019, when the first cases of COVID-19 were detected in Wuhan, China, healthcare systems across the world have been challenged to meet the demand for clinical care related to COVID-19 and non-COVID-19 health needs. In the initial stages of the pandemic, advice to stop disease transmission focused primarily on public health and hospital care. Guidance and support was provided to these health services, which were rapidly adapting to provide care to those needing urgent attention.^1^ Primary care also had a crucial role in responding to the COVID- 19 pandemic as the first point of patient contact and gatekeeper to secondary care.^2^^,^^3^ Services have had to react rapidly to provide care for both COVID-19 and non-COVID-19 patients while protecting all patients and professionals.^2^ Studies highlighted that remote consultations in primary care rapidly increased during the pandemic, changing management of patients.^4^

There is some understanding of the challenges facing primary care professionals (PCPs) during other pandemics. Previous work during influenza, Ebola, and severe acute respiratory syndrome pandemics showed that PCPs want to help in a pandemic. It also highlighted that they may struggle to implement new workflows and require additional training and equipment.^2^^–^5 This research was commonly retrospective, meaning reports were clouded by knowledge of how an outbreak evolved.

A few qualitative studies have explored the impact of caring for patients in the context of COVID-19.^5^^–^9 These studies have mainly been conducted in hospital settings and include views of hospital nurses on the impact of work on their mental health;^9^ hospital workers’ experiences of working during the pandemic;^6^^,^^8^ or nurses’ views of working with limited personal protective equipment (PPE).^7^ A limited number of studies report the views of PCPs but these are confined to a single country’s experiences.^4^^,^^10^^–^12 In this study this gap is addressed by investigating how PCPs in different European countries responded during the first peak of the COVID-19 pandemic, with the aim of understanding the key ingredients behind successful rapid transformation and adaptation of primary care to inform this ongoing crisis and any future crisis in care delivery.

**Table:** How this fits in

Previous qualitative studies exploring the experiences of healthcare professionals during the COVID-19 pandemic have mainly focused on secondary care. This study explored the experiences of primary care professionals (PCPs) on primary care transformation during the first peak of the COVID-19 pandemic in England, Belgium, the Netherlands, Ireland, Germany, Poland, Greece, and Sweden. PCPs described rapidly adapting to new circumstances by making decisions about how to transform primary care delivery for both patients with COVID-19 and patients with non-COVID-19 conditions, with limited training and resources. Flexibility and autonomy are necessary ingredients in primary care provision that should be preserved, coupled with provision of practical information on how to adapt services, ongoing training, and mental health support services for PCPs.

## METHOD

### Participants

Twenty countries from the authors’ existing clinical European primary care network (the Netherlands, Belgium, Denmark, Norway, UK, Spain, Greece, Germany, Poland, Hungary, Croatia, Ukraine, Moldova, Armenia, Georgia, Ireland, France, Romania, Sweden, and Switzerland) were sent an invitation to take part in the study. Ten countries expressed an interest. Eight countries were purposively selected, to get variation in the number of confirmed cases of COVID-19 (assessed in March 2020, see Supplementary Table S1), health system organisation (taking into account main differences pre-pandemic in relation to organisation of primary care, as presented in Supplementary Table S2), and geographical location in Europe. These countries included: England, Belgium, Ireland, the Netherlands, Germany, Poland, and Greece. In addition, Sweden was also included because of its different approach to lockdown. Denmark and Switzerland, who expressed an interest, were not included. Each country has a network coordinator who has access to a number of primary care sites. PCPs were recruited from these existing sites.

Convenience and purposeful sampling were used to recruit PCPs with a range of demographics and primary care experience. PCPs were invited to participate in the study by email or telephone. The aim was to recruit 80 PCPs (10 in each country).

### Interviews

Nine experienced qualitative primary care researchers completed interviews. All interviewers followed a topic guide (see Supplementary Box S1) on PCPs’ views on delivering care during the pandemic. Interviews took place by telephone, apart from in Greece (where they were face to face). All participants gave verbal or written consent to take part in the study. Interviews were audiorecorded and transcribed verbatim. Interviews were conducted in one of the main languages of each country and then translated into English, where relevant, to be analysed.

### Data analysis

All interviews were analysed by one of the authors, using a combination of deductive and inductive thematic analysis.^13^ Transcripts from England, Belgium, and the Netherlands were read line by line and data were coded into an a priori framework of 14 categories (see Supplementary Box S2), based on the topic guide and agreed by the core research team (deductive component). Data within each category was then coded line by line in order to create subcategories (inductive component). These were then grouped to form themes and subthemes, with a particular focus on key policy issues. A pragmatic approach was taken to data analysis; the order of analysis was dictated by the availability of translated interview data in English.

This thematic framework was then used to analyse data from other countries, and involved an iterative and consensus-based approach identifying similarities and differences among PCPs until data saturation was reached. The framework underwent substantial changes highlighting the variety of views and experiences not only across, but also within, countries. For example, in the initial interviews the ‘changes to roles’ mainly related to working in the ‘COVID hubs’, and this was reflected in the subthemes. Later on these were expanded to illustrate ‘changes to preexisting roles’.

At each stage of this process, data were discussed within the study team on a weekly basis. To further ensure rigour, the ongoing analysis was discussed within the multidisciplinary study team and all interviewers in each country on a monthly basis to ensure understanding of the local context, where relevant to interpreting findings. NVivo (version 12) was used to facilitate data analysis.^14^ This article adheres to the Consolidated Criteria for Reporting Qualitative Research (COREQ) reporting guideline.

## RESULTS

Eighty interviews were conducted between 2 April and 2 July 2020 and lasted between 17 and 86 min (mean 35 min). The timing of interviews in relation to lockdown restrictions in each country is summarised in Supplementary Table S1. Basic characteristics of participants are summarised in Table 1.

**Table 1.** Characteristics of participants by country

**Characteristic**	**England (*n*= 11)**	**Belgium (*n*= 10)**	**Netherlands (*n*= 10)**	**Ireland (*n*= 10)**	**Germany (*n*= 9)**	**Greece (*n*= 10)**	**Poland (*n*= 10)**	**Sweden (*n*= 10)**	**All countries (*n*= 80)**
Age, years, range (mean)	29–62 (47.3)	29–63 (44.0)	33–56 (45.8)	32–60 (43.3)	29–61 (43.2)	26–51 (39.8)	29–59 (49.2)	31–58 (43.5)	26–63 (44.5)
Sex, female, *n* (%)	8 (73)	5 (50)	6 (60)	6 (60)	5 (56)	8 (80)	9 (90)	7 (70)	54 (68)
GPs, *n*/ *N*	7/11	10/10	10/10	10/10	4/9	3/10	8/10	5/10	57/80
Nurses, *n*/*N*	4/11	N/A	N/A	N/A	1/9	4/10	2/10	4/10	15/80
Other healthcare professionals, *n*/*N*	N/A	N/A	N/A	N/A	4/9^a^	3/10^b^	N/A	1/10^c^	8/80
Experience, years, range	1–32	5–38	2.5–19	4–33	6–37	3–20	4–37	9–31	1–38
Tested for COVID-19 at time of interview, *n*/*N*	None	3/10	2/10	6/10	3/9	None	1/10	3/10	18/80

a *Two GP registrars; one physician assistant; one paediatrician working in primary care.*

b *One assistant nurse, one social worker, one paediatrician working in primary care.*

c *Nurse assistant (responsible for testing patients for COVID-19). N/A = not applicable.*

Four themes were identified:

1. transformation of primary care delivery and PCPs’ experiences of these changes;
2. PCPs’ sense of personal risk;
3. navigating a new relationship with patients; and
4. PCPs’ views of COVID-19 testing.

Given the depth of data gathered across countries, in this article only data on the first theme and its five subthemes are reported. The remaining themes will be reported in subsequent manuscripts. Key findings in relation to each of these subthemes is presented in Supplementary Table S3. Key information in relation to each country is presented in Supplementary Table S2 and additional quotes in Supplementary Table S4.

### Theme 1: Transformation of primary care delivery and PCPs’ experiences of these changes

#### Subtheme 1: managing patients with respiratory tract infection (RTI) symptoms

At first, PCPs in all countries tried to manage the majority of patients with RTI symptoms over the telephone, using history taking, follow-up, and safety netting. Care for patients needing face-to-face appointments was organised differently across countries. England, Belgium, the Netherlands, Germany, Sweden, and Greece set up processes to manage (suspected) COVID-19 patients in their own practice, which involved seeing them in a separate part of the surgery or at specific times to minimise risk.

With time, England, Belgium, and the Netherlands set up COVID hubs in certain cities (places where PCPs could send patients for examination and/or testing). PCPs welcomed them, but they also posed challenges and resulted in having to follow different protocols. PCPs in Belgium initiated and established COVID hubs themselves without any guidelines; and COVID hubs in England were often set up by different primary care organisations (clinical commissioning groups [CCG] and primary care networks [PCNs]):

*‘To make it more manageable they should be setting up these hubs … a lot quicker, they should be a lot more standardised but every CCG is doing it slightly differently and every PCN is doing things differently.’*

(GP, Participant [P]5, England)

In contrast, PCPs in Ireland stopped seeing patients face to face and all consultations were moved online, and patients were referred for testing by PCPs in drive-through testing centres.

Germany, Greece, and Sweden continued seeing patients with RTI in their practices throughout the pandemic and PCPs in Sweden described how they initially set up tents outside of the surgery, but with time, established more permanent areas for patient care. In contrast, patients in Poland who had symptoms of COVID-19 and were suspected of having contact with someone with COVID-19, were advised to contact hospitals or wards for infectious diseases. PCPs found it difficult at times to interpret guidance on this:

*‘Sometimes, when the symptoms are numerous and typical of a COVID-19 infection, we don’t even invite a patient in but we send him to the hospital straight away. In unclear cases, a doctor decides whether to examine a patient or not.’*

(GP, P7, Poland)

#### Subtheme 2: providing non-COVID-19 care

Initially, in all countries, the majority of conditions were managed by telephone. PCPs tried to assess what was urgent and required face-to-face care without much guidance; this appeared to result in variation across practices (see Supplementary Table S3).

With time, PCPs across all countries started to express their concern about ‘collateral damage’ resulting from routine care being postponed or limited, especially for chronic conditions. This made PCPs uncomfortable, and some realised that they would be dealing with the ‘backlog’ for a long time:

*‘I haven’t had a diabetes clinic for 3 months now, and that was of course frustrating; … I haven‘t caught up with my waiting list and seemingly won‘t have for all of this year.’*

(Nurse, P10, Sweden)

Countries differed in how they tried to maintain routine care. PCPs in most countries described their attempts of providing health checks and annual reviews remotely or in group format to help with a backlog; in contrast, PCPs in Belgium and Sweden suspended annual reviews and annual checks, respectively, for >70 year- olds. Some countries also focused on vulnerable patients by proactively calling them or increasing home visits. These decisions were initiated by each practice individually based on what they found most useful and feasible.

PCPs across all countries felt overwhelmed with constantly changing information from multiple official sources. However, they still reported lacking official training on PPE use, telephone triage, or practical information about how to organise or restart their care. PCPs in England, the Netherlands, and Poland highlighted that resuming care needed to be done in line with secondary care, allowing referrals for hospital investigations and with consideration for safety protocols.

#### Subtheme 3: resources to deliver primary care services during the pandemic: who pays?

PCPs in Belgium, the Netherlands, and Ireland, where GPs are paid for consultations, described the financial implications of the pandemic. Belgian and Dutch PCPs highlighted initial lack of clarity about whether telephone consultations would be reimbursed or paid for at the rate for face-to-face consultations; Belgian PCPs also set up and financed COVID hubs themselves initially. In Ireland, decreased workload, the hesitance of patients to pay for a telephone consultation, and the worry of PCPs that the patient may move to a different practice, caused financial concern:

*‘Nobody talks about the competition but it‘s always there … Patients might just leave one and go to the other practice and all that it has financial implications.’*

(GP, P2, Ireland)

Participants reported that lack of PPE or having to source and buy it without government support, sometimes at very high cost, was one of the main problems, coupled with a lack of clear guidance on when to use PPE or being told to ‘save it’:

*‘Until the end of March we didn’t have any suits … When the PPE suits arrived, they were very few, so if we used them, they would barely last a week.’*

(GP, P1, Greece)

The extent of provision of resources such as computers, webcams, and software allowing PCPs to provide remote care differed within and across countries, with some practices getting support from CCGs in England or the Narodowy Fundusz Zdrowia (National Health Fund) in Poland making it easier for PCPs to set up remote care. Practices had to cover the costs related to changing the layout of surgery buildings themselves.

#### Subtheme 4: remote care and dealing with uncertainty

All countries organised triage in order to prioritise and respond to patients’ queries. This was in contrast (apart from in the Netherlands) to an ‘open door policy’ that operated pre-pandemic (see Supplementary Table S2). PCPs across all countries described some limitations of remote care for both patients with and without RTI symptoms.

For patients with RTI symptoms, PCPs highlighted the difficulty in assessing whether and when patients will deteriorate. Limited knowledge and changing guidance on typical and atypical symptoms in the early stages of the pandemic was difficult to deal with:

*‘There’s no evidence base behind it — everyone’s just guessing … so you feel very unprofessional, you feel … the whole imposter syndrome comes right the way up. You just think, “Gosh, am I doing this right?”.’*

(GP, P3, England)

The majority of PCPs across all countries had limited experience with managing patients remotely before the pandemic. Not seeing their own patients and lack of visual clues meant that PCPs often worried about missing something important:

*‘Sometimes it’s hard to tell from a phone call if someone actually needs more attention or to be diverted to the hospital. Every choice we make is a risk.’*

(GP, P3, Greece)

In contrast, some PCPs in England and Sweden had experience with telephone and video consultations before the pandemic and found it easier to adjust to them. PCPs in Germany, England, Sweden, and Poland commented on wanting to continue with remote consultations for some patient queries in future but highlighted the difficulty of remote consultations for certain groups:

*‘I think it works with the younger group of patients … whereas I don’t think it works as well for the older generation. You can’t take away the benefits of sitting with a patient to assess them visually.’*

(Nurse, P6, England)

#### Subtheme 5: adjusting to roles and workloads and the importance of team work

PCPs described taking on additional tasks both formally, such as helping in setting up COVID hubs or acting as a triage person in the practice, and informally, by acting as a ‘counsellor’ to staff. These new roles at times also had an impact on PCPs’ workload; which had a negative impact on their mental health:

*‘Such an exhaustion, different from what we usually know, even though we have stressful and exhausting consulting hours, but that is another kind of exhaustion.’*

(GP, P8, Germany)

In contrast, some PCPs experienced a decrease in clinical workload and wanted to contribute more (see Supplementary Table S3). PCPs in Poland felt that they had been given a limited remit in relation to management of patients with RTI symptoms:

*‘Our role hasn’t been precisely defined so we continue caring for our patients … We are a bit left out.’*

(GP, P3, Poland)

In the context of these challenges, PCPs across all countries seemed to turn to their colleagues for moral support and by, for example, setting up daily team updates or using social media to share information and discuss patient cases (see Supplementary Table S3). Working together as a team was crucial, especially within their own practice but also with secondary care and other service providers. PCPs in Belgium and the Netherlands highlighted this in the context of GP surgeries working in groups, and some PCPs in Ireland and Belgium described how for the first time they had felt a sense of community among PCPs:

*‘In the practice, we work with four people so we could help each other. Like look there’s a new update on this subject, or have you seen that? Or I do it that way. So yes, you could inspire each other a little bit.’*

(GP, P2, Belgium)

Working well together and towards the same goal brought huge satisfaction and a sense of solidarity (see Supplementary Table S3):

*‘We see ourselves as all in this together, in the end, we are lucky to be able to have this job because patients allow us to have this job.* […] *As you know and I said it before, our competitive relationship with each other can make it difficult to collaborate. Nobody talks about the competition but it’s always there. In that people set-up practices beside each other and patients might just leave one and go to the other and all that it kind of has financial implications.’*

(P2, GP, Ireland)

## DISCUSSION

### Summary

To the author’s knowledge, this is the first pan-European qualitative study exploring views of PCPs during the first wave of the COVID-19 pandemic. As a result of the uncertainty surrounding diagnosis, management, and treatment of COVID-19 infection, PCPs were forced to rely on their own judgement and had to rapidly transform services to protect their patients and themselves. Despite being overwhelmed with guidance from multiple official sources, and ever-changing and sometimes contradictory information, they reported lacking access to practical training. As a result, PCPs turned to their colleagues for moral support and information to try to get used to remote care, and deal with uncertainty.

Key cross-country comparisons in relation to the formal roles of primary care were noted. There were similarities and differences in how countries responded to allocating roles in primary care during the pandemic. All countries quickly moved to providing the majority of consultations remotely. One of the key differences seemed to be whether countries set up places where PCPs could see suspected COVID-19 patients. All countries, apart from Poland, initially set up processes to manage the majority of (suspected) COVID-19 patients within general practices. This meant having to set up procedures to manage patients safely within practices, without much practical help. With time Belgium, the Netherlands, England, and Ireland organised COVID hubs that meant that PCPs had to, or chose to, take on additional roles in these settings, which affected workload. In addition, there were differences in who organised these hubs and how much involvement PCPs had in organising them. Hubs in England were organised by CCGs and PCNs, hubs in Belgium and the Netherlands were organised by GPs themselves from the ‘bottom-up’, and hubs in the Netherlands were organised by PCPs with assistance from decentralised municipal public health services (Gemeentelijke Gezondheidsdienst). It is important to point out that these different approaches resulted in variation in how hubs were organised. In contrast, in Poland most care related to COVID-19 was moved to hospitals or wards for infectious diseases.

### Strengths and limitations

To the author’s knowledge, this is the first qualitative study exploring the experiences of European PCPs on transformation of primary care during the first wave of the COVID-19 pandemic. This large, unique dataset provides valuable insights while also highlighting contextual differences in healthcare systems. Interviews were conducted in one of the main languages of each country, thus, allowing the collection of rich data. Despite a large number of interviews overall, the number of interviews in each country was relatively small with recruitment taking place from the authors’ existing network of practices, which may not represent the full picture of the situation in each country. However, extensive discussions with teams in each country aimed to facilitate understanding of the wider issues facing primary care in all the respective countries. Also, interviews took place at different time points thus making comparison between countries at times difficult.

### Comparison with existing literature

This study highlights a number of novel findings in relation to PCPs’ experiences of delivering care during the COVID-19 pandemic.

This study found that PCPs had to quickly adjust to providing remote care. For consultations with patients with respiratory and/or related COVID-19 symptoms this meant uncertainty in assessing symptoms remotely and for non-COVID-19 care it raised questions around minimising ‘collateral damage’. PCPs also highlighted that with time they became more confident in conducting remote consultations but factors such as not seeing their own patients or not being able to rely on visual cues meant that remote consultations and assessing who should be seen face to face still posed a challenge. This was in line with two previous studies exploring experiences of PCPs in England and Belgium, which also highlighted the importance of these factors.^4^^,^^11^ PCPs in the present study also highlighted the need to consider patients’ needs and preferences when assessing the need for a remote consultation and described that certain patient groups may not be able or want to engage with this way of providing care, thus highlighting additional factors that PCPs find important.

In line with another study in Belgian primary care,^11^ PCPs in the present study reported the need to attend to patients with non-COVID-19 concerns and worry about ‘collateral damage’. The present study further highlights the importance of this concern as it was shared by PCPs in different European countries. PCPs also reported receiving numerous official guidance but they often commented on limited access to practical information on how to manage patients with COVID-19 symptoms and how to maintain or restart routine care. This meant that PCPs and teams made these decisions themselves, leading to variation in care.

Finally, this study found that PCPs showed great resilience, despite difficulties. Although daily communication within teams brought a sense of solidarity and togetherness, for some individuals it was not sufficient. The negative impact of working in the pandemic on mental health is now well documented;^14^^–^16 however, there has been limited focus on this topic in primary care. The current study highlights the resilience of PCPs while also drawing attention to the mental health needs of PCPs.

### Implications for practice

The implementation of telemedicine in the initial stages of the pandemic was necessary to allow patients to receive care at home thus avoiding the spread of COVID-19. However, it also introduced the need for risk assessment,^17^ which PCPs found difficult. Gaining experience with remote consulting can help as it was shown that PCPs with pre-pandemic experience of remote consultations found adjusting to the new situation easier. It is important to highlight that other factors may be important as well. With the volume and complexity of issues patients present with increasing, remote management can become more time consuming, riskier, and less satisfying.^4^ This may also be the case when patients with non-COVID-19 symptoms start seeking care as PCPs have to deal with the backlog resulting from postponing non-COVID-19 care during periods of lockdown.

In addition, this study highlighted that PCPs also wanted to take into consideration the preferences and needs of patients, as some patients (for example, older adults) may still prefer face-to-face consultations. Therefore, PCPs need ongoing training in delivering remote consultations related to both COVID-19 and non-COVID-19 care. This is crucial, especially in the context of the backlog from the first wave, winter season pressures, and having to reconfigure services again.^18^ In later stages of easing lockdown, consideration of whether to maintain or decrease remote consultation, and crucially how this is implemented, will be very important as well. It is important PCPs have a say in how they want to manage this process to be able to adapt guidelines to their local contexts (for example, areas with higher and lower COVID-19 rates), clinical need, and patients’ needs and preferences. Others also highlighted that the model of remote consulting may need to evolve.^4^ However, if PCPs are to continue or restart seeing some patients face to face, they also need to have an adequate supply of PPE and resources to adapt practices to provide care safely.

Although team working and autonomy of PCPs facilitated rapid transformation of care delivery, there was also a need for standardised information focused on the practical steps needed to change processes. Each country and policy organisations such as the European Centre for Disease Prevention and Control^19^^,^^20^ and World Health Organization^21^ issued guidance, but PCPs were not always aware of these and did not perceive them as sufficient in organising their care. This shows the need for provision of practical information, ideally from one central source in each country, preferably organisations serving PCPs (for example, in the UK the Royal College of General Practitioners or in Poland, Polskie Towarzystwo Medycyny Rodzinnej [Polish Association of Family Medicine]). This needs to be balanced with the need for autonomy of PCPs in being able to adapt this information to their local contexts. This will be key for responding to future health emergencies and pandemics.

Future research could also investigate the views of PCPs and members of key policy organisations in their respective countries and at European level to identify how they could work together. This needs to be planned and coordinated with secondary and community care to work together more efficiently as recent national lockdowns across Europe may mean another wave of cancellation of hospital tests and procedures, putting the burden on primary care again to manage these patients.^22^

PCPs can also be at risk of mental ill health and compassion fatigue, which will become more pertinent with the prolonged demands as a result of the current and future waves.^11^ PCPs need access to appropriate mental health services to be able to continue providing patient care. A summary of key recommendations for practice is presented in Box 1.

**Box 1.** Summary of recommendations in relation to key issues

**Key issues**	**Recommendations**
Organisation of care for COVID-19 and non-COVID	Provide overarching practical guidance on how to transform services from one central source Provide clinicians with clear information about how they can maintain non-COVID-19 care in order to mitigate the secondary impacts of the pandemic Ensure primary care representation at policy level and engagement with local primary care champions
Resources	Provide personal protective equipment with training on how and when to use Provide clear information on reimbursement process and financial resources to support service redesign Provide information technology infrastructure to facilitate remote care
Remote care and dealing with uncertainty	Provide ongoing training in adapting remote care to respond to patients’ needs and emerging evidence on COVID-19 Provide and encourage clinicians to use resources to support their mental health and facilitate resilience Acknowledge that uncertainty is a common and natural reaction when working in a pandemic and that clinicians are not able to do their jobs as normal
Adjusting to new processes, roles, and workloads, and the importance of team work	Encourage primary care teams to share advice and resources both within and between teams to keep up with guidance when guidance is changing quickly Consider impact of changes in workload and its impact on mental health

In conclusion, PCPs rapidly transformed primary care delivery despite a number of challenges. Primary care needs clear representation at policy level and engagement with local primary care champions to continue having a vital role in responding to this and future pandemics. This is needed to facilitate coordinated access to practical information on how to adapt services, ongoing training in delivering remote consultations, and access to mental health services for PCPs. Preservation of autonomy and local responsiveness of primary care are critical to preserve the ability for rapid transformation in any future crisis of care delivery.
